# A Sustainable Solution for the Adsorption of C.I. Direct Black 80, an Azoic Textile Dye with Plant Stems: *Zygophyllum gaetulum* in an Aqueous Solution

**DOI:** 10.3390/molecules29204806

**Published:** 2024-10-11

**Authors:** Chaimaa Haoufazane, Fatima Zaaboul, Hanae El Monfalouti, Nada Kheira Sebbar, Mohamed Hefnawy, Abderrahim El Hourch, Badr Eddine Kartah

**Affiliations:** 1 Laboratory of Plant Chemistry, Organic and Bioorganic Synthesis, Faculty of Sciences, Mohammed V University in Rabat, 4 Avenue Ibn Battouta, BP, P.O. Box 1014, Rabat 10090, Morocco; shaimaa.haoufazane@gmail.com (C.H.); n.sebbar@uiz.ac.ma (N.K.S.); b.kartah@um5r.ac.ma (B.E.K.); 2 Laboratory of Materials, Nanotechnologies and Environment, Chemistry Department, Faculty of Sciences, Mohammed V University of Rabat, Rabat 10090, Morocco; zaaboul.fatima00@gmail.com (F.Z.); elhourch@yahoo.fr (A.E.H.); 3 Laboratory of Organic and Physical Chemistry, Applied Bioorganic Chemistry Team, Faculty of Sciences, Ibnou Zohr University, Agadir 80000, Morocco; 4 Department of Pharmaceutical Chemistry, College of Pharmacy, King Saud University, Riyadh 11451, Saudi Arabia; mhefnawy@ksu.edu.sa

**Keywords:** *Zygophyllum gaetulum*, plant, stem, adsorption, C.I. Direct Black 80, textile dye, biosorbent, kinetics, isotherm

## Abstract

The presence of pollutants in water sources, particularly dyes coming by way of the textile industry, represents a major challenge with far-reaching environmental consequences, including increased scarcity. This phenomenon endangers the health of living organisms and the natural system. Numerous biosorbents have been utilized for the removal of dyes from the textile industry. The aim of this study was to optimize discarded *Zygophyllum gaetulum* stems as constituting an untreated natural biosorbent for the efficient removal of C.I. Direct Black 80, an azo textile dye, from an aqueous solution, thus offering an ecological and low-cost alternative while recovering the waste for reuse. The biosorbent was subjected to a series of characterization analyses: scanning electron microscopy (SEM), thermogravimetric analysis (TGA), Brunauer–Emmett–Teller (BET) method, X-ray diffraction (XRD), and infrared spectroscopy (IR) were employed to characterize the biosorbent. Additionally, the moisture and ash content of the plant stem were also examined. The absorption phenomenon was studied for several different parameters including the effect of the absorption time (0 to 360 min), the sorbent mass (3 to 40 g/L), the pH of the solution (3 to 11), the dye concentration (5 to 300 mg/L), and the pH of the zero-charge point (2–12). Thermodynamic studies and desorption studies were also carried out. The results showed that an increase in plant mass from 3 to 40 g/L resulted in a notable enhancement in dye adsorption rates, with an observed rise from 63.96% to 97.08%. The pH at the zero-charge point (pHpzc) was determined to be 7.12. The percentage of dye removal was found to be highest for pH values ≤ 7, with a subsequent decline in removal efficiency as the pH increased. Following an initial increase in the amount of adsorbed dye, equilibrium was reached within 2 h of contact. The kinetic parameters of adsorption were investigated using the pseudo-first-order, pseudo-second-order and Elovich models. The results indicated that the pseudo-first-order kinetic model was the most appropriate for the plant adsorbent. The isotherm parameters were determined using the Langmuir, Frendlich, Temkin, and Dubinin–Radushkevich models. The experimental data were more satisfactory and better fitted using the Langmuir model for the adsorption of dye on the plant. This study demonstrated that *Zygophyllum gaetulum* stems could be employed as an effective adsorbent for the removal of our organic dye from an aqueous solution.

## 1. Introduction

Dyes are organic compounds used to impart color to materials such as textiles, paper, and plastics [1]. Used in sectors such as textiles, cosmetics, and scientific research, their chemical structures, particularly their chromophores, determine properties such as their solubility and stability [1,2,3]. Around 800,000 tons of dyes are produced worldwide every year, including 200,000 tons for textiles [4,5]. Azo dyes, which account for 60–70% of all dyes used in the textile industry, present environmental risks as 15–20% of them are released into the environment [6,7]. Their complex structures make it difficult for them to biodegrade, with potential carcinogenic and toxic effects. 

The importance of this problem is accentuated by the central role of water, an essential resource for life. As well as being a universal solvent, water regulates temperature, enables chemical reactions, and maintains biodiversity in ecosystems [8,9,10]. However, the contamination of water resources by textile dyes considerably reduces its availability, contributing to the global water crisis and directly threatening public health and the balance of ecosystems. The world currently has around four billion people under water stress, representing almost two-thirds of the world’s population [11]. Such dyes, which are chemical complex compounds, can enter water bodies through intentional discharges, inadequate treatment, or accidental spills [5,12].

Indeed, it is very important to develop effective methods for removing dyes from water sources and industrial wastewater. Several treatment methods have been developed to effectively remove dyes. These include reverse osmosis, precipitation, ultrafiltration, ion exchange, nanofiltration, coagulation, flocculation, flotation, electrodialysis, and adsorption [8,13,14,15,16,17]. However, many of these techniques have disadvantages in terms of selectivity, secondary waste generation, and cost.

Of all the methods mentioned above, adsorption has established itself as a cost-effective and practical process, making it one of the most favored treatments for removing dyes [18,19]. However, to address the limitations of traditional adsorption techniques, biosorption, which involves the use of biomaterials as absorbents, has emerged as a promising alternative. This approach utilizes biological materials such as algae, fungi, or plants as biosorbents for enhanced dye removal and a reduced environmental impact [20,21,22]. 

Among these biosorbents, several plants and agricultural residues have been studied, including Sargassum (seaweed) [23], rice husk [24], peanut shells [25], wheat straw [26], neem leaves [27], coconut fiber [28], banana leaves [29], mango peel [30], coconut shell [31], and orange peel [32]. These natural materials offer an ecological solution for wastewater treatment and their adsorption efficiency relies on the presence of various functional groups such as hydroxyls, carboxyls, and amine groups, which interact with contaminants [33].

*Zygophyllum gaetulum*, known locally as “Al Berraya” or “Al Aggaya”, is a medicinal plant endemic to the Moroccan Sahara, particularly in the Drâa region [34]. While numerous studies have highlighted its hypoglycemic, antifungal, anti-inflammatory, and anti-diarrheal properties [35], no previous research has examined its potential as an adsorbent for dye removal.

The present study was a first exploration of the use of *Zygophyllum gaetulum* stems as natural, unmodified biosorbents for the removal of synthetic dyes from aqueous solutions. The use of removed stems highlights not only the durability of this approach but also its potential for low-cost, environmentally friendly applications. Unlike other studies, which have often relied on chemically modified adsorbents, this research focused on the use of unprocessed biomass, ensuring a more sustainable and feasible alternative for large-scale implementation.

In the present work, we focus on the removal of C.I. Direct Black 80, a widely used dye in the textile industry known for its colorfastness and resistance to fading but which is also an environmentally persistent textile dye. Although other hazardous dyes exist, Direct Black 80 was chosen because of its complex anionic structure and widespread use in industry, making its effective disposal a significant challenge. This study analyzed the key parameters affecting the adsorption process, such as the initial dye concentration, contact time, temperature, pH, and adsorbent dosage. In addition, we assessed the recyclability of the biosorbent, evaluating its potential for reuse in multiple cycles. Adsorption kinetics and equilibrium isotherms were also examined to better understand the adsorption mechanism, contributing to the development of sustainable and reusable biosorption technologies.

## 2. Results and Discussion

### 2.1. Characterization of the Biosorbent

#### 2.1.1. Brunauer–Emmett–Teller (BET) Analysis

A comprehensive examination of the surface and porosity characteristics of the *Z. Gaetulum* stem powder through Brunauer–Emmett–Teller (BET) analysis offers a vital insight into the assessment of surface and porosity characteristics for biosorption applications. The fundamental principle of the BET method is the measurement of the multilayer physisorption of non-corrosive gases (typically nitrogen) on solid materials [36].

The results of the BET analysis, along with other surface measurements, are summarized in Table 1. The BET isotherm was employed to ascertain the surface area (SBET-multipoint), which was found to be 1.71 m^2^/g. The textural properties were analyzed according to the model of Barrett, Joyner, and Halenda (BJH), which yielded a cumulative pore volume of approximately 0.0026 cm^3^/g and an average particle size of 3.51 nm. These findings indicate the presence of a well-defined mesoporous structure with minimal microporosity. The Langmuir surface area of 2.334 m^2^/g, indicative of monolayer adsorption on a homogeneous surface, provides a comprehensive conclusion to these results.

**Figure 1:**
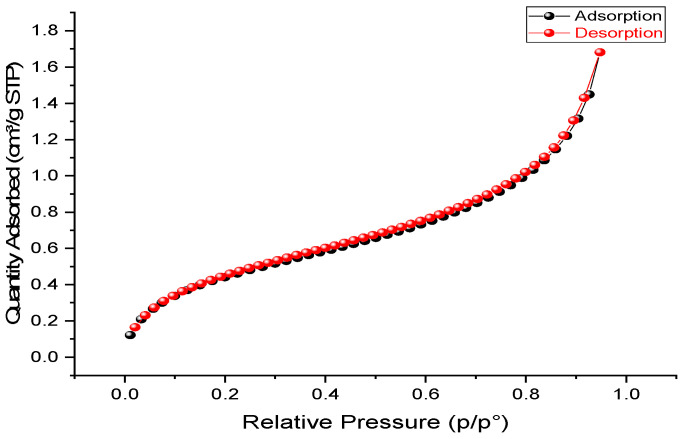
N_2_ adsorption–desorption isotherm of *Z. Gaetulum* stem powder.

Figure 1 illustrates the N_2_ adsorption–desorption isotherm of *Z. Gaetulum* stem powder. According to the IUPAC classification, it is distinctly assigned to type IV with a negligible H3 hysteresis loop. This evidence corroborates the mesoporous nature of our biosorbent.

#### 2.1.2. Fourier-Transform Infrared Spectroscopy (FTIR)

**Figure 2:**
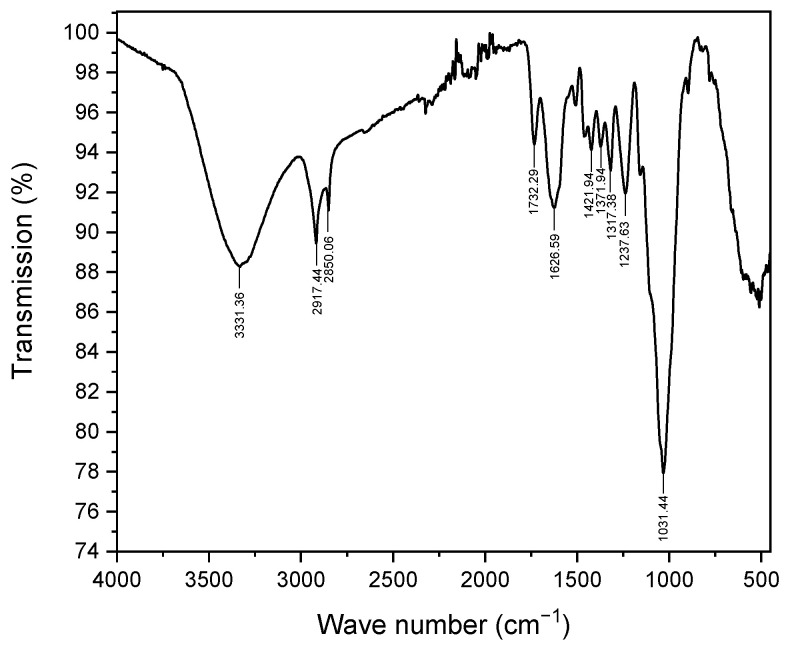
FTIR spectra of *Zygophyllum gaetulum* stem powder.

Figure 2 illustrates the FTIR analysis of *Zygophyllum gaetulum* stem powder before the adsorption of the C.I. Direct Black 80 dye. This provides a comprehensive understanding of its phytochemical composition, which primarily comprises lignin, cellulose, and hemicellulose. The broad band at 3331.36 cm^−1^ is attributed to hydroxyl stretching vibrations (-OH), which are characteristic of cellulose and hemicellulose due to their polysaccharide structures, as well as the hydroxyl groups present in lignin [37]. The peaks at 2917.44 cm^−1^ and 2850.06 cm^−1^ indicate the presence of aliphatic C-H stretching vibrations, in particular methyl (CH_3_) and methylene (CH_2_) groups, which are integral to the aliphatic chains of hemicellulose and the complex structure of lignin [38]. The small peak at 1732.29 cm^−1^ indicates the presence of carbonyl groups (C=O), typical of ketones and aldehydes present in hemicellulose and lignin [38,39]. In addition, the vibration observed at 1626.59 cm^−1^ suggests the presence of C=C bonds, which are indicative of unsaturated aromatic structures inherent to lignin. The bands at 1421.94 cm^−1^, 1371.94 cm^−1^, and 1317.38 cm^−1^ correspond to the C-H bending of alkyl groups, which are present in both the polysaccharide chains of cellulose and hemicellulose as well as in lignin. The peak lengths of 1237.63 cm^−1^ and 1031.44 cm^−1^ indicate C-O stretching vibrations, suggesting the presence of ether and alcohol groups. These groups are characteristic of both glycosidic bonds in cellulose and hemicellulose as well as ether bonds in lignin [40].

The presence of these functional groups explains the high adsorption capacity of our biomass. Hydroxyl, carbonyl, and aromatic groups are particularly effective as they interact with the negatively charged sulfonate groups of the Direct Black 80 anionic dye, forming electrostatic interactions and hydrogen bonds. In addition, π-π interactions between lignin’s aromatic rings and the dye molecules further enhance the adsorption process. Collectively, these functional groups create a network of binding sites that contribute to the efficient removal of Direct Black 80 from aqueous solutions.

#### 2.1.3. Thermal Properties

**Figure 3:**
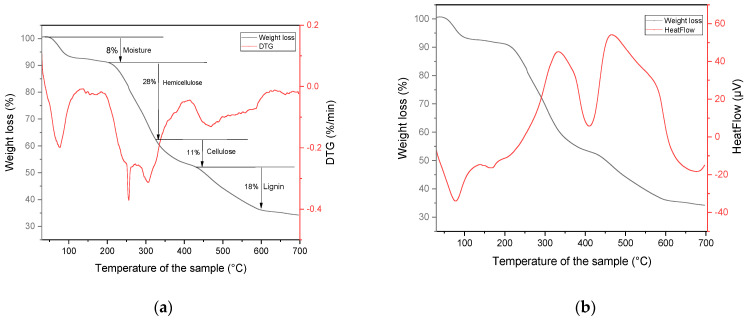
(**a**) TGA/DTG analysis; (**b**) TGA/DSC analysis of *Zygophyllum Gaetulum* stems.

Thermogravimetric (TGA), differential thermogravimetric (DTG), and differential scanning calorimetric (DSC) analyses of the *Zygophyllum gaetulum* stem powder provided detailed insight into its thermal decomposition and heat flux characteristics (Figure 3). The TGA curve revealed the existence of four distinct stages of weight loss. An initial weight loss of approximately 8% occurred below 100 °C due to the loss of moisture and volatile components. A correspondingly small DTG peak served to confirm the evaporation of moisture. This stage was typical of lignocellulosic materials whereby water and light volatiles were released at low temperatures [41]. 

The subsequent notable weight loss (28%) occurred between 200 and 300 °C and was attributed to the thermal degradation of hemicellulose. Hemicellulose undergoes decomposition at a lower temperature than cellulose and lignin. The first major peak in the DTG curve indicated the maximum rate of weight loss due to hemicellulose decomposition.

A notable weight loss of 11% was observed between 300 °C and 400 °C, which correlated with the thermal degradation of cellulose. The DTG peak in this region corresponded to the maximum degradation rate of cellulose.

Finally, lignin is more thermally stable than cellulose and hemicellulose and decomposes over a wide temperature range. In our sample, this occurred above 400 °C, with a weight loss of 18%. The DTG curve displayed several minor peaks in this region, which was indicative of the complex nature of lignin decomposition [41]. 

The DSC curve corroborated these findings with an endothermic peak below 100 °C, indicative of moisture evaporation; a minor endothermic event between 100 and 250 °C, associated with hemicellulose decomposition; a prominent exothermic peak between 250 and 410 °C, reflective of cellulose degradation; and a wide exothermic peak between 410 and 650 °C, which represented the complex decomposition of lignin [42].

#### 2.1.4. X-ray Diffraction Analysis (XRD)

**Figure 4:**
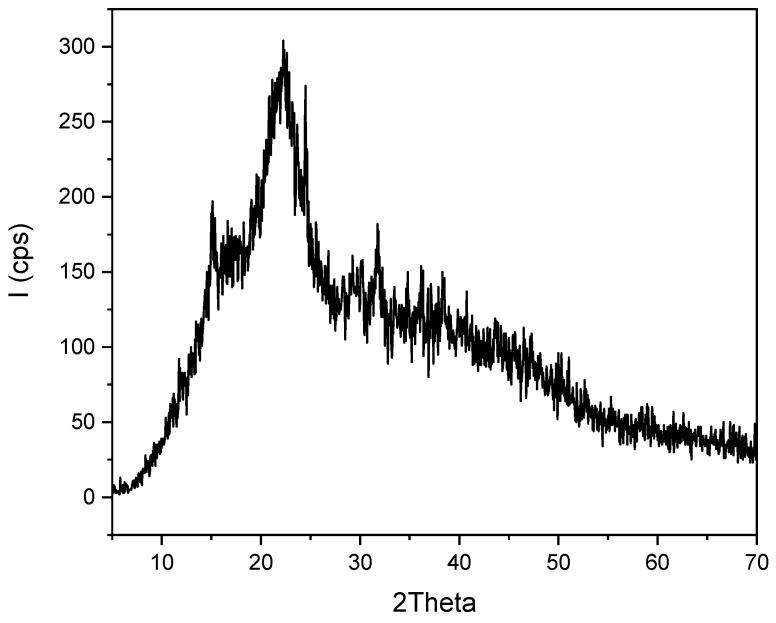
XRD diffractogram of *Zygophyllum gaetulum* stems.

Figure 4 illustrates the X-ray diffraction (XRD) pattern of *Zygophyllum gaetulum* stem powder. The presence of an intense peak at approximately 2θ ≈ 23°, with a value of around 300 cps, indicates the existence of a crystalline phase. This can be attributed to the properties of crystalline cellulose, which is a principal constituent of plant cell walls [43]. 

The different orientations of cellulose crystals, as well as the presence of lignin and hemicellulose, which are amorphous and semi-crystalline compounds, can be achieved by the observation of other peaks present at different values of 2θ. Furthermore, an amorphous fraction above 2θ ≈ 40° is discernible by the presence of lignin and hemicellulose. The existence of both was additionally corroborated by FTIR and ATG analyses. 

#### 2.1.5. Morphology

Scanning electron microscopy (SEM) coupled with energy-dispersive X-ray (EDX) spectroscopy was employed to examine the morphological structure and the elements present within the *Zygophyllum gaetulum* stem structure.

**Figure 5:**
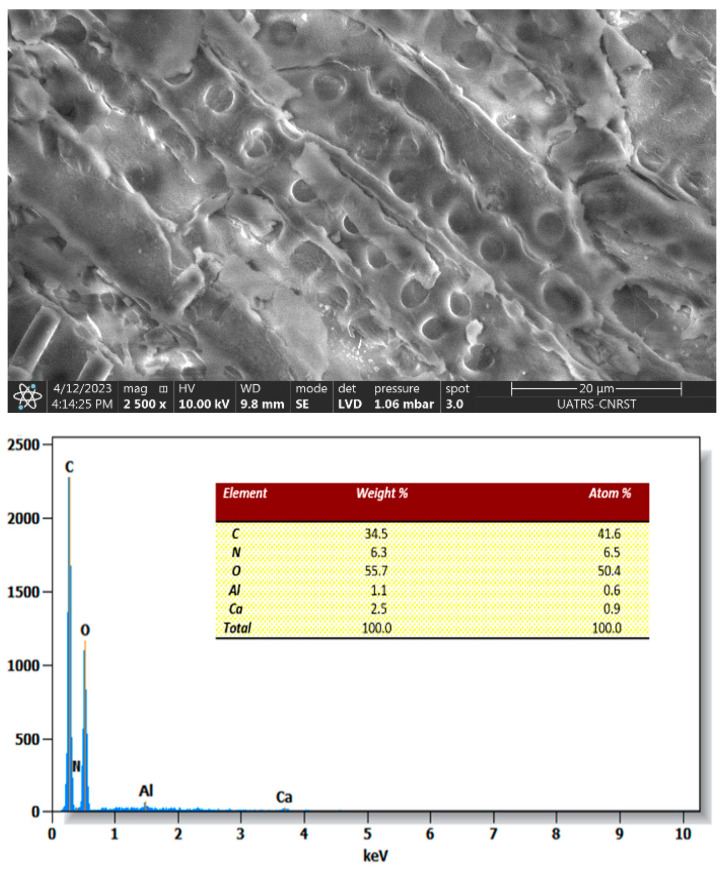
SEM images and EDX spectra of *Zygophyllum gaetulum* stem powder before adsorption.

Figure 5 shows an image of the *Zygophyllum gaetulum* stems, which exhibited a structure reminiscent of small sticks with high porosity. The outer surface was characterized by the presence of cavities with mesoporous structures, which facilitated the adsorption of dyes.

#### 2.1.6. Zero-Charge Point

The influence of pH-dependent variations in the surface charge on adsorption processes is of paramount importance. These variations exert a selective influence on the affinity of the adsorbent for anions or cations that is contingent upon the prevailing acid–base conditions.

For this purpose, we have determined the zero-charge point with a view to enhancing our understanding of the underlying mechanism. The pHpzc is defined as the pH at which the total charge on the adsorbent surface becomes zero [20]. 

**Figure 6:**
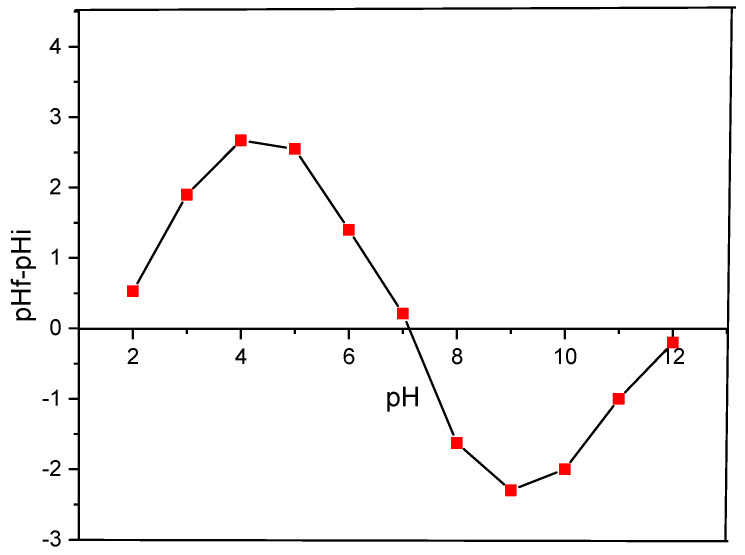
Determination of pHpzc of *Zygophyllum gaetulum* stems.

The pHpzc of *Zygophyllum gaetulum* stems was found to be 7.12 as shown in Figure 6. At a pH of the solution below the PZC (point of zero charge), the adsorbent surface acquires a positive charge due to the protonation of the acid groups present in the material. Simply expressed, the adsorbent surface becomes cationic, favoring the adsorption of anions. Conversely, when the pH of the solution exceeds the pH PZC, oxygen-containing acid groups on the surface are ionized or dissociated. In short, the adsorbent surface adopts an anionic charge, showing a preference for cation adsorption [44].

### 2.2. Effect of pH of Dye Solution

Dye removal by *Zygophyllum gaetulum* stems was studied at room temperature (T = 25 °C) over a pH range of 3 to 11, including the natural pH (pH = 7) of the blue–black dye solution. This was achieved through the utilization of a biosorbent dosage of 20 g/L and an initial dye concentration of 50 mg/L. 

**Figure 7:**
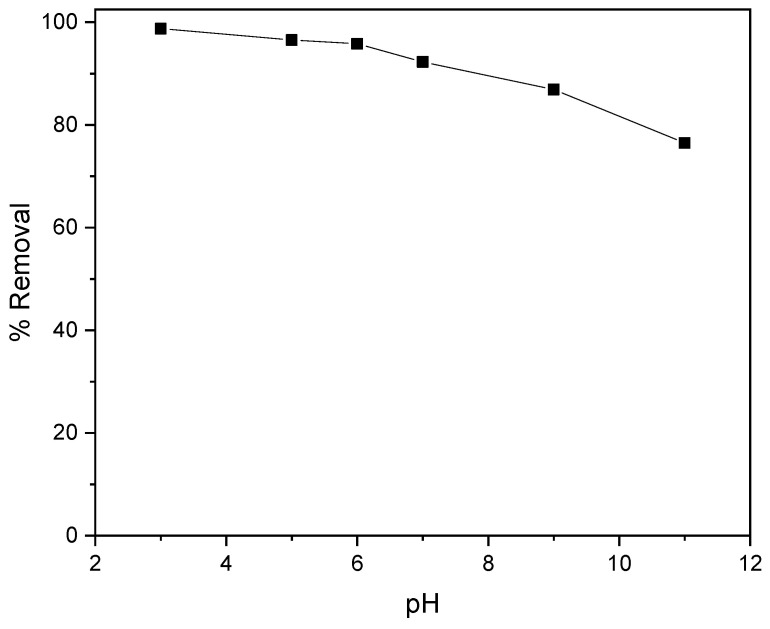
Effect of initial pH on the removal efficiency of C.I. Direct Black 80 dye on *Zygophyllum Gaetulum* stems (C_0_ = 50 mg.L^−1^, t = 2 h30, V = 25 mL, biosorbent dosage = 20 g.L^−1^, T = 25 °C).

The impact of the pH on the removal of C.I. Direct Black 80 by the stems is illustrated in Figure 7. To attain the requisite pH, sulfuric acid (H_2_SO_4_) (0.1 M) and sodium hydroxide NaOH (0.1 M) were employed to calibrate the solution. 

The adsorption of the anionic dye C.I. Direct Black 80 by the biosorbent was significantly affected by pH variations and linked to the zero-charge point (pHpzc) of our biosorbent. The percentage of dye removal was highest for pH values ≤ 7. The results show that at pH = 3, better removal was achieved. This is explained by the positive charge of the biosorbent surface at pHs below the pHpzc (7.12), favoring a strong electrostatic attraction with anionic ions of the dye such as sulfonates (-SO_3_^−^). At pH = 3, the protonation of the biosorbent’s functional groups, such as that of hydroxyls (-OH), reinforced this positive charge, optimizing adsorption by attraction between opposite charges. On the other hand, at pH values above 7.12, the biosorbent surface became negatively charged, resulting in electrostatic repulsion with the dye’s anionic ions, reducing adsorption efficiency. This repulsion, combined with competition from OH- ions in the solution, explains the progressive decrease in the percentage of dye removal at higher pH values.

### 2.3. Effect of Bioadsorbent Dosage

One of the key factors in the adsorption process lies in the mass of the adsorbent [45]. This experiment aimed to impact of adsorbent dosage on the elimination efficacy of C.I. Direct Black 80 by *Zygophyllum Gaetulum* stem powder.

**Figure 8:**
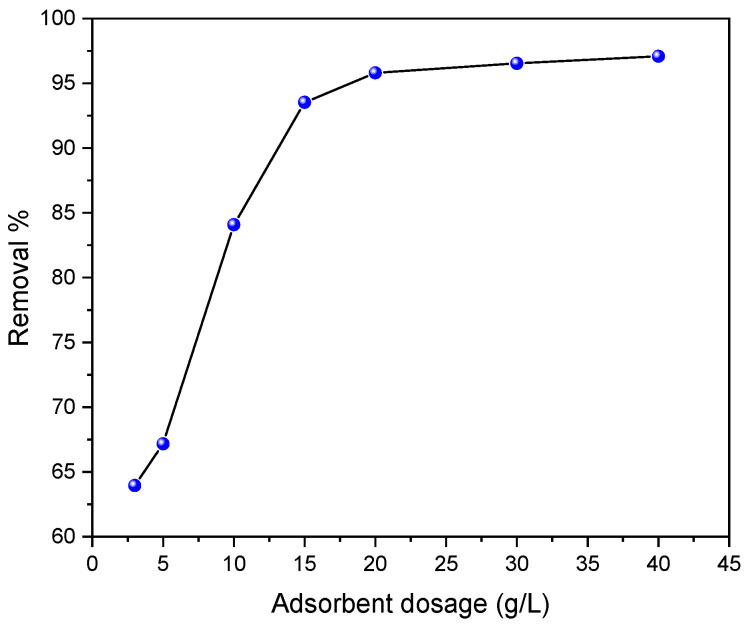
Effect of adsorbent dose on the removal efficiency of C.I. Direct Black 80 dye on *Zygophyllum gaetulum* stems (C_0_ = 50 mg.L^−1^, t = 150 min, V = 25 mL, T = 25 °C).

The effect of the *Z. Gaetulum* stem powder dosage on the percentage removal and adsorption capacity of C.I. Direct Black 80 is represented in Figure 8. The results demonstrate that a mass of 3 g/L of the biomass achieves a removal rate of 63.96% while a 20 g/L dose of the bioadsorbent results in a removal rate of 96%.

An increase in the dose of *Zygophyllum gaetulum* stems leads to an increase in the percentage of C.I. Direct Black 80 elimination. 

This is due to an increase in the number of available adsorption sites, which is directly proportional to the quantity of the adsorbent applied. The increase in the dye removal percentage, as a function of different adsorbent masses, can be attributed to the expansion of adsorbent surfaces, thus promoting an increase in the number of adsorption sites available for the adsorption process. These findings align with those previously reported in the literature [46].

### 2.4. Adsorption Kinetics

The kinetic parameters of adsorption describe the mechanism and the rate at which an adsorbate is adsorbed onto a surface. Thus, their study is essential to our understanding of adsorption. Various kinetic models can be applied to describe this process.

The analysis of kinetic parameters for the process of the adsorption of CI Direct Black 80 by *Zygophyllum gaetulum* stem powder was carried out by fitting experimental data to three distinct kinetic models: the pseudo-first-order (PFO) model, the pseudo-second-order (PSO) model, and the Elovich model. 

According to Lagergren, pseudo-first-order kinetics prove that the speed of the sorption process is directly proportional to the dye concentration, being particularly relevant for relatively low concentrations [47]. The pseudo-first-order kinetic model is formulated by the following Equation (1):

(1) $$ln(qt)=ln(qe)-\frac{k_{1}t}{2.303}$$

The pseudo-second-order kinetic model is founded on the hypothesis that the rate-limiting step is chemical sorption, or chemisorption. This allows behavior to be predicted over the entire adsorption range [48]. The pseudo-second-order kinetic model is formulated by the following Equation (2):

(2) $$\frac{t\;}{qt}=\frac{1}{h}+\frac{t}{qe}\;$$

Here, $h=k_{2}{q_{e}}^{2}$.

*h*: Initial sorption rate.
*q_e_*: Amount of the dye adsorbed at equilibrium (mg/g).
*k*_2_: Equilibrium rate constant of the pseudo-second-order model (g/mg min).

(3) $$q_{t}=\frac{1}{\;\beta }\;ln(\alpha \beta t+1)\;$$

*q_t_* is the amount of the adsorbate adsorbed at time *t*.
*α* is the initial adsorption rate.
*β* is the desorption constant.
*t* is the time.

The study experiment involved a variation in the contact time between the dye and the adsorbent, with a range of 0 to 360 min. The initial dye concentration was 50 mg/L (25 mL) and the adsorbent concentration was 20 g/L. The solutions were stirred with a magnetic stirrer at room temperature and the dye pH.

The Elovich model, first developed by Zeldovich and Elovich, is commonly used to describe the kinetics of chemisorption (or chemical adsorption). This model assumes that the adsorption process involves a chemical reaction between the adsorbate and the adsorbent surface, which leads to the formation of a monolayer [49]. 

**Figure 9:**
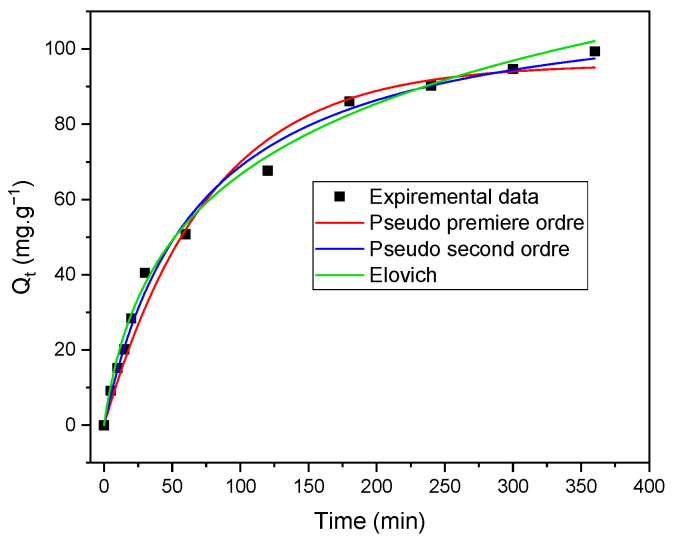
Non-linear fitting of kinetic models for the adsorption of C.I. Direct Black 80 dye on *Zygophyllum gaetulum* stems (C_0_ = 50 mg.L^−1^, T = 25 °C, V = 25 mL, adsorbent dosage = 20 g.L^−1^).

The data on the adsorption of C.I. Direct Black 80 on *Zygophyllum gaetulum* stem powder provide significant kinetic information. The experimental data were fitted to three distinct kinetic models: the pseudo-first-order (PFO) model, the pseudo-second-order (PSO) model, and the Elovich model. The PFO model was identified as the most appropriate, exhibiting a notable coefficient of determination (R^2^) of 0.999, as illustrated in Table 2. Figure 9 shows the non-linear fitting of kinetic models for the adsorption of C.I. Direct Black 80 dye on *Zygophyllum gaetulum* stem powder. Indeed, the adsorption of the dye showed rapid initial adsorption over the initial 25 min, followed by the progressive saturation of the active sites. The PFO model describes this rapid adsorption mechanism, mainly regulated by physical interactions such as hydrogen bonds between the dye and hydroxyl groups present in cellulose and hemicellulose. However, at longer contact times, the PSO model points out that a chemical component may be involved. The Elovich model, with an R^2^ of 0.972, highlights a certain heterogeneity of adsorption sites, probably linked to the more complex structure of lignin, which offers varied sites in terms of adsorption energy. These results indicate that at longer times, more complex and varied interactions influence adsorption. So, although physical interactions predominate overall, adsorption in this system is also influenced by the heterogeneous structure of the biomass, particularly lignin, which may play a longer-term role in overall adsorption efficiency.

### 2.5. Adsorption Isotherms

The adsorption isotherm describes the relation with the adsorbate in the phase surrounding the adsorbent and the adsorbate adsorbed on the surface of the adsorbent at equilibrium and constant temperature.

In the present study, the isotherms used to determine the effect of dye concentration on our phenomenon were the Langmuir, Freundlich, Temkin, and Dubinin–Radushkevich isotherms.

The equation for Langmuir’s isotherm is probably the most widely known and applied equation to describe adsorption equilibrium. 

Langmuir’s adsorption model provides an approach to asymptotic monolayer surface coverage as the partial pressure of the adsorbate moves toward saturation. This model is used in ideal conditions and supposes monolayer adsorption, i.e., each active site on the surface is occupied by a single molecule of the adsorbent [50]. The equation is stated as follows:

(4) $$Qe=\frac{Qm\;K_{L}\;Ce}{1+K_{L}\;Ce}$$

Here,

*Q_m_* is the maximum sorption capacity (mg/g).
*K_L_* is the Langmuir constant (L/mg)

The Freundlich isotherm is widely used to study multi-monolayer adsorption systems on heterogeneous surfaces, in contrary to the Langmuir isotherm, which assumes monomolecular adsorption on a homogeneous surface [51]. The equation is defined as follows:

(5) $$Q_{e}=K_{F}{Ce}^{\;\frac{1}{n}}$$

*K_F_*: Freundlich constant (mg^1−(1/n)^(dm^3^)^1/n^g^−1^); *n*: heterogeneity factor.

The Temkin isotherm model is an adsorption model that considers the interactions between molecules adsorbed on a solid surface. It implies, with an increasing adsorbent surface coverage, that the heat of adsorption of all molecules decreases linearly. Furthermore, the model considers that adsorption is marked by a uniform distribution of binding energies reaching a maximum binding energy [50]. The equation is expressed by the following formula:

(6) $$Q_{e}=\frac{RT}{b}Log\left(K_{T}C_{e}\right)$$

*b*: adsorption constant (J/mol).
*K_t_*: Temkin isotherm constant (L/mg).

The Dubinin–Radushkevich (D-R) isotherm is deployed to describe adsorption on heterogeneous surfaces, determining whether adsorption is physical or chemical based on the average adsorption potential energy (*E*). Considering the distribution of adsorption energies over the available sites, this model is particularly suitable for systems where adsorption does not follow a strictly monomolecular pattern [52,53].

(7) $$Q_{e}=Q_{d}\exp(-K_{ad}{\left(RTln\left(1+\frac{1}{C_{e}}\right)\right)}^{2})$$

*Q_d_* is the theoretical maximum isotherm saturation capacity (mg/g).
*K_ad_* is the Dubinin–Radushkevich isotherm constant related to the sorption energy (mol^2^/J^2^).

The average adsorption potential energy (*E_p_*) provides an understanding of the nature of the adsorption (chemisorption, ion exchange, or physisorption) and is calculated from the following equation:

(8) $$E_{p}=\frac{1}{\sqrt{2K_{ad}}}$$

Physical adsorption: $E_{p}<8.0\;kJ/mol$; ion exchange: $8.0\;kJ/mol\leq E_{p}<16.0\;kJ/mol$; chemical adsorption: $E_{p}\geq 16.0\;kJ/mol$.

**Figure 10:**
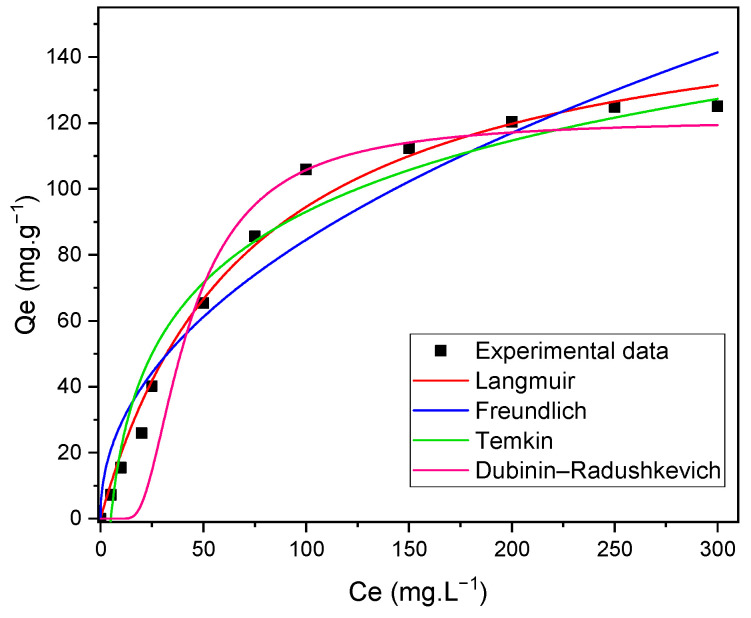
Non-linear fitting of adsorption isotherms of C.I. Direct Black dye 80 on *Zygophyllum gaetulum* stems (T = 25 °C, t = 150 min, V = 25 mL, adsorbent dosage = 20 g.L^−1^).

The overall results, summarized in Table 3, suggest that the Langmuir model is the most appropriate to describe adsorption in our system, with both a higher coefficient of determination (R^2^ = 0.98) compared to the others (0.94, 0.91, and 0.89) as well as a lower SSE and RSME, offering the best fit to the experimental data (Figure 10).

Adsorption in this experiment followed a monomolecular adsorption pattern on a homogeneous surface. The maximum capacity of 163.23 mg/g suggests that the hydroxyl groups of cellulose and hemicellulose form hydrogen bonds with C.I. Direct Black 80 while lignin contributes π-π stacking interactions. This model aptly describes the homogeneous nature of adsorption sites, reflecting essentially physical interactions.

The Freundlich model indicates the heterogeneity of adsorption sites, probably due to the complex structure of lignin. Lignin, with its phenolic groups and branched chains, creates adsorption sites that are varied in terms of energy, unlike cellulose and hemicellulose, which offer more homogeneous sites. However, this heterogeneity is less marked than expected, which explains the less accurate fit compared with the Langmuir model. 

Temkin’s model, which considers repulsive interactions between adsorbed molecules, suggests that these interactions are present but insignificant in this system.

The Dubinin–Radushkevich (D-R) model confirms the physical nature of adsorption, with a very low adsorption energy (E = 0.019 kJ/mol), linked to Van der Waals interactions and hydrogen bonds, consistent with the nature of the adsorption sites provided by cellulose and hemicellulose. Although this model shows some site heterogeneity, its fit remains inferior to that of the Langmuir model, confirming that adsorption mainly follows a homogeneous mechanism.

### 2.6. Thermodynamic Studies 

Thermodynamic parameters such as Gibbs free energy (ΔG°), enthalpy (ΔH°), and entropy (ΔS°) play a central role in understanding adsorption mechanisms, enabling us to assess both the spontaneity and exothermic or endothermic nature of the process as well as structural modifications to the system. The following procedure was used to determine the adsorption equilibrium constant (*K_ad_*) and associated thermodynamic parameters [54]. The *K_ad_* was obtained from the Langmuir equilibrium parameter *K_L_*, previously converted from unit (L/mg) to unit (L/mol) using Equation (9):

(9) $$K_{L}\left(L/mol\right)=K_{L}\left(L/mg\right)\times 1000(mg/g)\times M(g/mol)$$

*M* is the molecular weight of C.I. Direct Black 80 (g/mol).

Subsequently, *Kad* was estimated using Equation (10):

(10) $$K_{ad}=K_{L}\left(L/mol\right)\times C_{ref}(mol/L)\times \frac{1}{\gamma }$$

*γ* is the activity coefficient calculated as a function of ionic strength.
*C_ref_* is the reference molar concentration.

Under conventional conditions, *C_ref_* is fixed at 1 mol/L, making *K_ad_* the same as *K_L_*. However, in certain practical circumstances, when the dye’s solubility in water is less than 1 M, the reference concentration is adjusted to the saturation concentration (*C_s_*). C.I. Direct Black 80, for example, has a water solubility of around 30 g/L (0.033 mol/L).

In this context, the adsorption equilibrium constant (*K_ad_*) was calculated by applying Equation (10), taking the saturated solution as the reference concentration, i.e., *C_ref_* = *C_S_* (mol/L). The activity coefficient (*γ*) was determined using the Davis relationship [55], ensuring accurate evaluation as a function of the system’s ionic conditions.

**Figure 11:**
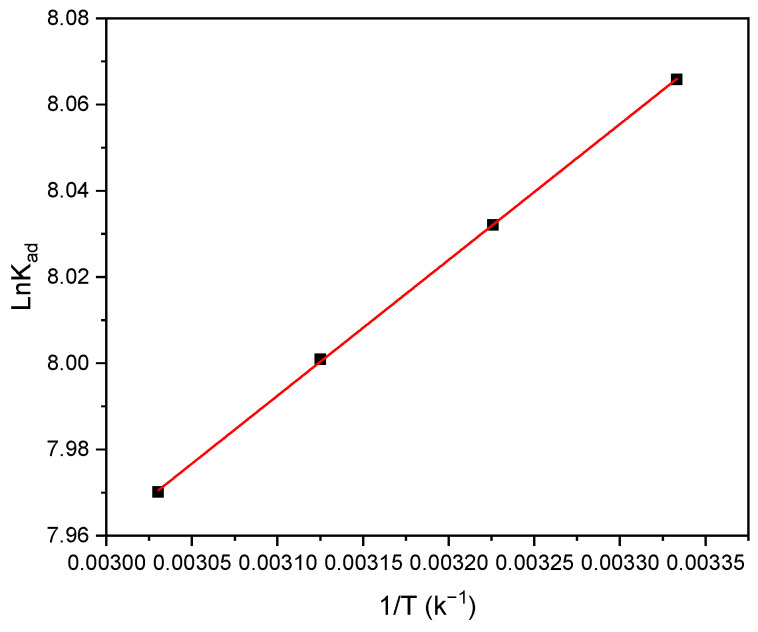
Plot: ln(K_ad_) vs. 1/T for determination of thermodynamic parameters of C.I. Direct Black 80 adsorption on *Zygophyllum gaetulum* stems.

By plotting *ln(K_ad_)* as a function of 1/T and applying the Van’t Hoff Equation (12) (Figure 11), it is possible to determine ΔH° and ΔS° from the slope and y-intercept, respectively. ΔG°, meanwhile, is obtained for each temperature using Equation (11):

(11) $$\Delta G{}^{\circ}=-RT\;ln(K_{ad})$$

(12) $$Ln\;K_{ad}=\frac{-\Delta H{}^{\circ}}{RT}+\frac{\Delta S{}^{\circ}}{R}$$

*K_ad_* is the equilibrium constant of adsorption.
Δ*G°* is the standard Gibbs free energy (J/mol).
Δ*H°* is the standard enthalpy (J/mol).
Δ*S°* is the standard entropy (J/K.mol).
*R* is the perfect gas constant (8.314 J/K.mol).
*T* is the absolute temperature (in Kelvin).

The thermodynamic parameters obtained for the adsorption of C.I. Direct Black 80 on *Zygophyllum gaetulum* stems are grouped in Table 4 and indicate a spontaneous, exothermic process. Indeed, a Δ*G°* value below 0 kJ/mol and a ∆*H°* value below 40 kJ/mol indicates physical adsorption whereas Δ*G°* values between −80 and −400 kJ/mol and *∆H°* values above 40 kJ/mol shows that chemical adsorption has occurred [52]. 

The negative values of Δ*G°* (−20.117 to −21.867 kJ/mol) show that adsorption is thermodynamically favorable although spontaneity decreases slightly with an increasing temperature, which is typical of an exothermic process. The low value of Δ*H°* (−2.619 kJ/mol) confirms the exothermic nature and suggests physisorption, characterized by weak interactions such as Van der Waals forces. The increase in entropy (Δ*S°* = 58.33 J/mol.K) suggests increased disorder at the solid–liquid interface, probably due to the release of water molecules or other species during adsorption, as confirmed by the FTIR results and the mass loss observed by TGA.

### 2.7. Desorption Studies 

**Figure 12:**
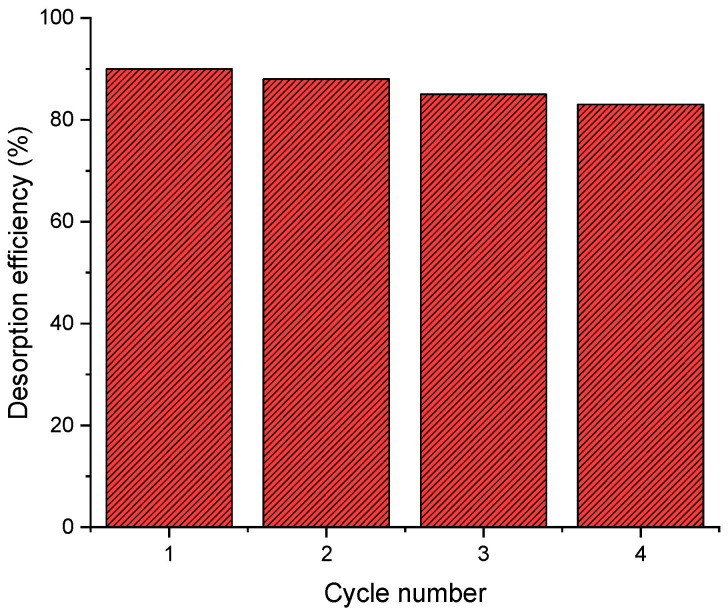
Desorption of C.I. Direct Black 80 from *Zygophyllum gaetulum* stems.

The results show that the desorption of C.I. Direct Black 80 dye by *Zygophyllum gaetulum* stems in NaOH solution (0.1 M) is extremely efficient, with an initial desorption rate reaching 90% in the first cycle (Figure 12). This is due to the effect of NaOH, which modifies the pH of the system above the zero-charge point (pHpzc) of the adsorbent, giving its surface a negative charge. This creates an electrostatic repulsion with the anionic dye, facilitating its release into the desorption solution.

In the second and third cycles, desorption rates were maintained at high levels, reaching 88% and 85%, respectively, demonstrating the adsorbent’s ability to be regenerated and reused efficiently. The consistency of the results over the cycles highlights the robustness of the adsorbent and the ability of NaOH to restore active sites, enabling adsorption–desorption to continue without major disruption to the overall performance.

Even after four cycles, the desorption rate remained above 80%, confirming the viability of the adsorbent in repeated applications. These results are in line with those of other studies where strong bases, such as NaOH, were used for the desorption of dyes and heavy metals, particularly due to their ability to break electrostatic interactions and effectively regenerate adsorbents [56].

### 2.8. Morphology after Adsorption

**Figure 13:**
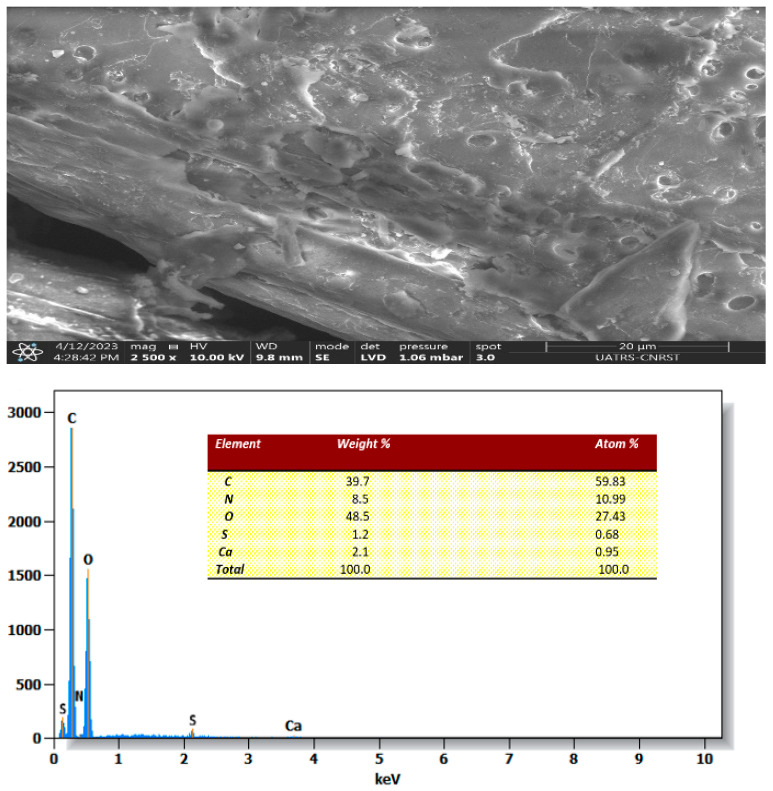
SEM images and EDX spectra of *Zygophyllum gaetulum* stem powder after adsorption (C_0_ = 50 mg.L^−1^, T = 25 °C, t = 150 min, V = 25 mL, adsorbent dosage = 20 g.L^−1^).

After the adsorption of the C.I. Direct Black 80 dye (as shown in Figure 13), a notable alteration in the structure of the biomass surface was observed. It became denser and exhibited a reduction in porosity. The surfaces of the *Zygophyllum gaetulum* stems became markedly slick as the C.I. Direct Black 80 dye was captured in the pores.

The chemical composition of the powder was determined through EDX analysis following the adsorption process. The appearance of sulfur and the increase in nitrogen after this process, as shown in Figure 13, suggest that the C.I. Direct Black 80 dye was adsorbed onto the powder.

### 2.9. FTIR after Adsorption

**Figure 14:**
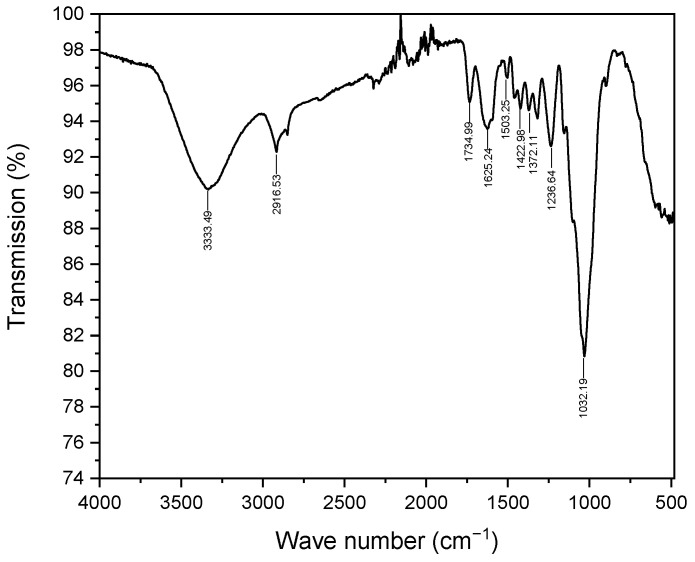
FTIR spectra of *Zygophyllum gaetulum* stem powder after adsorption.

After the adsorption of the C.I. Direct Black 80 dye (Figure 14), slight changes were observed in the FTIR spectrum, indicating specific interactions between the functional groups of the biosorbent and the dye. The broad band at 3333 cm^−1^, attributed to elongation vibrations of the O-H groups, showed a slight attenuation, which could have indicated the formation of hydrogen bonds between the biosorbent’s hydroxyl groups and the dye’s anionic groups such as sulfonates (-SO_3_^−^). The band at 1625 cm^−1^, associated with O-H vibrations or aromatic C=C bonds present in lignin, showed a change in intensity, which may have indicated interaction with azo groups (-N=N-) in the dye. The bands at 1503, 1422, and 1372 cm^−1^, corresponding to aromatic vibrations and C-H deformations in lignocellulosic structures, remained unchanged, confirming that the biosorbent structure remained intact after adsorption. Finally, the band at 1032 cm^−1^, previously attributed to C-O elongation vibrations, characteristic of glycosidic bonds in cellulose and hemicellulose and ether bonds in lignin, showed modifications that could have reflected interactions with the dye’s C-N bonds. This attribution is supported by the detection of nitrogen in EDX analysis after adsorption. Our results were consistent with observations reported by Enache et al. [54], who have demonstrated that dyes interact differently with lignocellulosic components. When interacting with cellulose, hydrogen bonds are mainly formed, corresponding to physical interactions dominated by Van der Waals forces. In contrast, lignin tends to establish more complex bonds with the functional groups of dyes, where electrostatic interactions, notably Coulomb forces, can play a predominant role. This distinction between the types of bonds and forces observed in our study, notably between hydrogen bonds and those involving the dye’s C-N groups, confirms the importance of specific interactions between biomass components and the C.I. Direct Black 80 dye. Overall, these results, confirmed by the Langmuir isotherm and pseudo-first-order kinetic studies, suggest that despite the presence of electrostatic interactions and hydrogen bonds, the adsorption mechanism is mainly governed by physisorption, as evidenced by the low adsorption energy values.

### 2.10. Comparative Study

A comparative study was conducted to evaluate the efficacy of this bioadsorbent in removing azo dye in comparison to other bioadsorbents (Table 5). The results show that our biomass, *Zygophyllum gaetulum* stems, is an effective adsorbent for the removal of the azo dye C.I. Direct Black 80, with a removal rate of 97.08%. These findings suggest that *Zygophyllum gaetulum* stems may be a promising option among the bioadsorbents studied for dye treatment.

## 3. Materials and Methods

### 3.1. Materials

Sigma-Aldrich (Saint Louis, MO, USA) supplied C.I. Direct Black 80 (C_36_H_23_N_8_Na_3_O_11_S_3_), sulfuric acid (H_2_SO_4_), and analytical-grade sodium hydroxide (NaOH) for this study. All chemicals used were of reagent grade and were used without further purification. Bi-distilled water was used exclusively for the preparation of all aqueous solutions.

### 3.2. Biosorbent Preparation

The biomass of *Zygophyllum gaetulum*, which was utilized in this study, was gathered in June 2022 from Foum Zguid, a town located in the southeastern region of Morocco’s Tata Province within the Souss-Massa area. 

In this study, only the discarded plant stems were used. The stems were cut into segments and underwent thorough washing with tap water to eliminate any dirt present. Subsequently, they were macerated with bi-distillated water for 24 h to eliminate chlorophyll and other pigments responsible for the plant’s coloration. Following maceration, the stems were dried in an oven at 90 degrees Celsius for 12 h. Once dried, they were ground using a household grinder and sieved through a 200 µm diameter stainless steel sieve to obtain the desired particle size.

### 3.3. Analysis of Moisture and Ash Content in the Bioadsorbent

In this study, the moisture and ash content of bioadsorbent were calculated using the methods described. After subjecting a 1 g sample of the plant material to drying at 110 °C over 24 h, the sample was left to cool and stabilize in a desiccator before being weighed to establish the dry weight. The sample was then calcined at 600 °C over 3 h to measure ash content. The calcination process effectively removed organic matter and volatile compounds, leaving behind the inorganic residue known as ash. After cooling in a desiccator, the sample was re-weighed.

The percentage of moisture content was calculated as follows:

(13) $$\%H=\frac{m_{1}-m_{2}}{m_{1}}\times 100\;$$

Upon performing the calculations, a moisture content of 10.41% was obtained, indicating the proportion of water present in the original sample.

Similarly, the ash content was calculated using the following formula:

(14) $$\%Ash=\frac{m_{3}}{m_{1}}\times 100$$

Here, *m*_3_ is the sample weight after calcination, *m*_2_ is the sample weight after drying, and *m*_1_ is the initial sample weight. 

The calculated ash content was found to be 16.52%, indicating the percentage of inorganic residue remaining after the removal of organic components.

### 3.4. Particle Size Analysis: Granulometric Study

The size distribution of a plant powder was examined, focusing on particles at 200 µm using a sieve. Particle size plays a critical role in biosorbent adsorption efficiency. Smaller particles offer larger surface areas and more active sites, leading to higher adsorption capacities and faster kinetics [62]. Conversely, larger particles have reduced surface areas, resulting in lower adsorption capacities [63].

### 3.5. Characterization

To comprehensively characterize *Zygophyllum gaetulum* stem biosorption capacity, a multifaceted approach was adopted. Fourier-Transform Infrared (FTIR) spectroscopy was employed to elucidate the plant material’s chemical composition, pinpointing functional groups responsible for biosorption interactions. The KBr pellet technique was utilized to record the Fourier-Transform Infrared (FTIR) spectra of the samples. This was accomplished on an FTIR spectrometer, covering the spectral range of 4000–400 cm^−1^ using Bruker (Billerica, MA, USA) Alpha Platinum-ATR instrument. X-ray diffraction (XRD) analysis provided insights into its crystalline structures, shedding light on potential binding sites in the 2θ range of 5–70°. XRD analysis was conducted by the Shimadzu (Tokyo, Japan) XDR-6100 instrument. Thermal stability and decomposition behavior were assessed through thermogravimetric analysis (TGA), differential thermogravimetric (DTG) analysis, and differential scanning calorimetry (DSC) using the Setaram Labsys TM Evo instrument (1F), running in a range of temperatures from 20 °C to 700 °C at a flux of air of 45 mL.min^−1^ and a heating rate of 10 °C.min^−1^. Scanning electron microscopy (SEM) was utilized to explore its surface morphology, revealing structural attributes conducive to biosorption. Furthermore, energy-dispersive X-ray (EDX) spectroscopy was employed to conduct a quantitative analysis of the elemental composition. SEM/EDX was conducted using the JEOL (Peabody, MA, USA) JSM-7600F instrument. Quantification of surface area and porosity was achieved via Brunauer–Emmett–Teller (BET) analysis using TriStar II Plus de Micromeritics Version 3.00.

### 3.6. Dye

**Figure 15:**
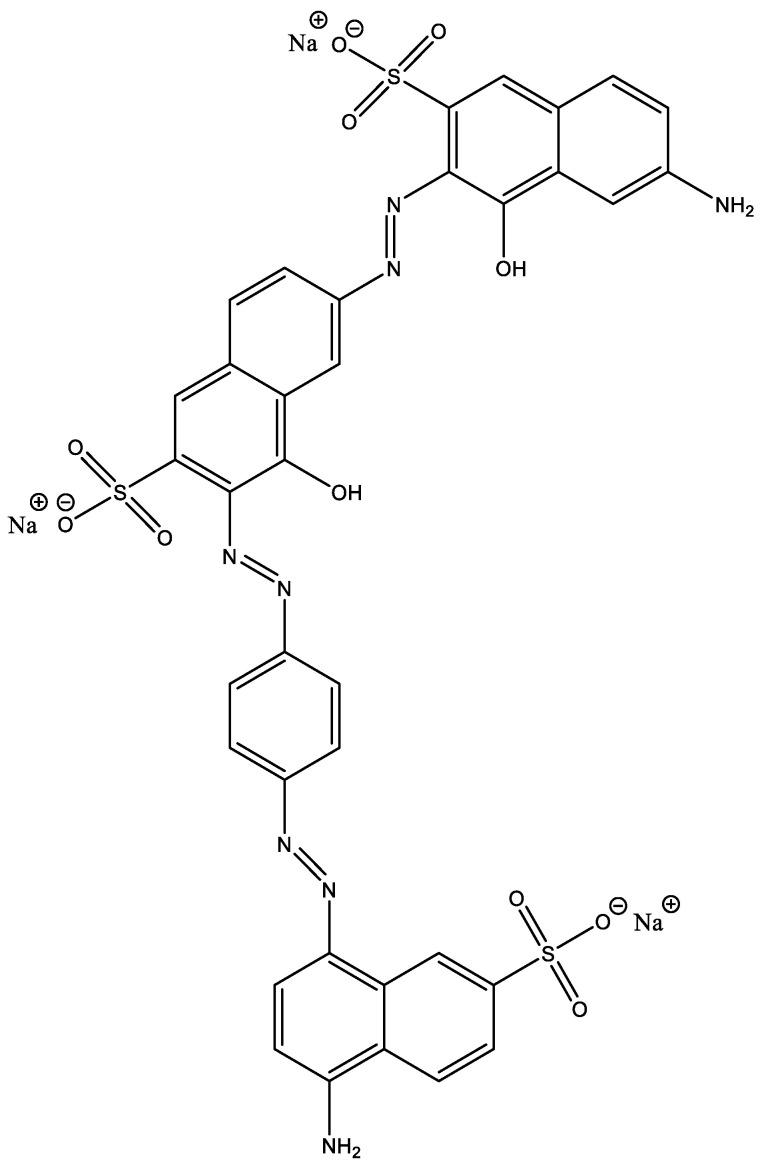
Chemical structure of C.I. Direct Black 80.

C.I. Direct Black 80, a member of the direct dye class, is categorized as an anionic dye (Figure 15). It possesses a molecular weight of 908.8 g/mol. Furthermore, this dye demonstrates a maximum wavelength (λ_max_) of 599.8 nm. The characteristics of C.I. Direct Black 80 are regrouped in Table 6.

### 3.7. Batch Adsorption Tests

Experimental procedures were conducted employing a 50 mg/L initial dye solution prepared within a 1 L flask. Varied parameters were explored: biosorbent mass (3 to 40 g/L), encompassing solution pH (ranging from 3 to 11), initial dye concentrations (ranging from 5 to 300 mg/L), and contact times (ranging from 0 to 360 min). pH adjustments utilized H_2_SO_4_ (0.1 M) and NaOH (0.1 M). Biosorption trials incorporated 0.5 g of biosorbent powder into 25 mL dye solution in an Erlenmeyer flask, followed by stirring at 300 rpm with the orbital shaker (Edmund Buhler GmbH, Bodelshausen, Germany). After the biosorption period, centrifugation at 1000 rpm for 10 min was conducted and the resulting supernatant was analyzed.

At a wavelength of 599.8 nm, the concentration of the solution was measured using a UV/VIS spectrophotometer, SP-UV1100 DLAB.

The removal rate % was determined using the following equation:

(15) $$\%Removal=\frac{C_{i}-C_{f}}{C_{i}}\times 100\;$$

Here, $C_{i}$ is the initial concentration (mg/L) and $C_{f}$ is the final concentration (mg/L).

Adsorption capacity at time *t*, *qt* (mg/g), was then calculated using the following formula:

(16) $$q_{t}=\frac{C_{i}-C_{t}}{m}\times V\;$$

Here,

$C_{t}$ = the concentration (mg/L) at any time t.
*V* (L) = the volume of the solution.
*m* (g) = the mass of the adsorbent.

Adsorption capacity at equilibrium, *q_e_* (mg/L), was calculated thus:

(17) $$q_{e}=\frac{C_{i}-C_{e}}{m}\times V\;\;$$

Here, *Ce* (mg/L) is the equilibrium concentration.

### 3.8. Desorption Studies

To evaluate the regeneration capacity of *Zygophyllum gaetulum* stem adsorbent after adsorption of C.I. Direct Black 80 dye, a desorption protocol was used. After each adsorption cycle, the saturated adsorbent was recovered by filtration and then immersed in a solution of NaOH (0.1 M). The volume of desorption solution used was 25 mL. The adsorbent was stirred with the orbital shaker (Edmund Buhler GmbH) (300 rpm) at room temperature for 2 h to enhance desorption of the adsorbed dye. After agitation, the adsorbent was recovered by vacuum filtration, rinsed with distilled water to remove NaOH residues, then dried at 70 °C for 12 h.

This process was repeated over several cycles to determine the adsorbent’s ability to be regenerated and reused. The concentration of dye in the desorption solution was measured by the UV/VIS spectrophotometer SP-UV1100 DLAB after each cycle to assess desorption efficiency. The % desorption (*DE%*) was calculated according to the following equation:

(18) $$DE\%=\frac{C_{d}\times V_{d}}{q_{e}\times m}\times 100$$

$C_{d}$ is the dye concentration in the desorption solution (mg/L).
$V_{d}$ is the volume of the desorption solution (L).
$q_{e}$ is the amount of dye adsorbed by the adsorbent (mg/g).
m is the mass of adsorbent used (g).

## 4. Conclusions

This study demonstrated that the discarded *Zygophyllum gaetulum* stems, as untreated natural biosorbents, are effective in removing the azo dye C.I. Direct Black 80 from aqueous solutions. Characterization analyses, including scanning electron microscopy (SEM), thermogravimetric analysis (TGA), infrared spectroscopy (IR), X-ray diffraction (XRD), and the Brunauer–Emmett–Teller (BET) method, provided essential information on the structure and physico-chemical composition of the biosorbent, including on the presence of cellulose, hemicellulose, and lignin, which are responsible for its adsorbent capacity. These compounds facilitate interactions with the dye via physisorption mechanisms, as confirmed by isotherms, kinetics, and thermodynamic studies. Adsorption parameters, such as the adsorbent mass, contact time, initial dye concentration, and solution pH, showed a significant impact on the efficiency of the adsorption process. Increasing the mass of the adsorbent achieved an adsorption rate of up to 97.08% at a concentration of 40 g/L, with maximum adsorption at acidic pHs (pH ≤ 7), promoting electrostatic attraction between the positively charged surface of the adsorbent and the anionic ions of the dye.

Kinetic studies revealed that the pseudo-first-order model best described the adsorption process, suggesting a predominance of physical interactions. Langmuir isotherms confirmed monolayer adsorption on homogeneous sites while thermodynamic studies indicated a spontaneous and exothermic process typical of physisorption. Finally, desorption studies showed that the adsorbent can be regenerated and reused over four cycles with a desorption efficiency of over 80%, demonstrating the viability of this biosorbent for repeated applications.

In brief, this study demonstrated that *Zygophyllum gaetulum* stems possess effective adsorbent properties for the removal of organic dyes in aqueous solutions. These findings pave the way for potential applications in the treatment of industrial effluents containing azo dyes, offering an economic and environmentally and sustainably viable alternative for water remediation.

## Figures and Tables

**Table 1:** Adsorptive and structural characterization of *Z. Gaetulum* stem powder.

Surface Area	
Single-point surface area at p/p° = 0.299756172	1.57 m^2^/g
BET surface area	1.71 m^2^/g
Langmuir surface area	2.33 m^2^/g
t-Plot external surface area	2.10 m^2^/g
BJH adsorption ^1^	1.68 m^2^/g
BJH desorption ^1^	1.87 m^2^/g
D-H adsorption ^1^	1.65 m^2^/g
D-H desorption ^1^	1.69 m^2^/g

^1^ Cumulative pore surface area ranging in size by 1.7000 nm and 300.0000 nm in diameter.

**Table 2:** Adsorption kinetics: parameters.

Models	Parameter	Value
Pseudo-first-order	*Qe* (exp) (mg.g^−1^)	97.61
	*Qe* (cal) (mg.g^−1^)	95.47 ± 2.84
	*K_1_* (min^−1^)	0.01303 ± 0.00135
	R^2^	0.999
	SSE	174.69
	RMSE	4.18
Pseudo-second-order	*Qe* (exp) (mg.g^−1^)	97.61
	*Qe* (cal) (mg.g^−1^)	116.08 ± 3.41
	*K_1_* (min^−1^)	0.000125 ± 0.000015
	R^2^	0.993
	SSE	91.56
	RMSE	3.03
Elovich	*α* (mg.g^−1^ min^−1^)	2.5253 ± 0.0478
	*β* (g.mg^−1^)	0.0338 ± 0.0003
	R^2^	0.972
	SSE	15,875.12
	RMSE	3.99

**Table 3:** Langmuir, Freundlich, Temkin, and Dubinin–Radushkevich isotherm parameters.

Isotherm	Parameter	Value
Langmuir	*Qm* (mg.g^−1^)	163.23 ± 7.55
	*K_L_* (L.mg^−1^)	0.013 ± 0.002
	R^2^	0.98
	SSE	304.42
	RSME	5.52
Freundlich	*K_F_* (mg.g^−1^)	9.8072 ± 2.9379
	1/*n*	0.4678 ± 0.0269
	R^2^	0.94
	SSE	1587.43
	RSME	12.62
Dubinin–Radushkevich	*Kad* (mol^2^/kJ^2^)	1379.53 ± 32.58
	*qd* (mg/g)	121.20 ± 0.47
	R^2^	0.89
	SSE	114,201.21
	RSME	10.67
Temkin	*K_T_* (L.g^−1^)	0.1989 ± 0.0064
	*B* (J.mol^−1^)	0.2018 ± 0.0024
	R^2^	0.91
	SSE	94,873.78
	RSME	9.76

**Table 4:** Thermodynamic parameters for C.I. Direct Black 80 adsorption on *Zygophyllum gaetulum* stems.

Dye	T (K)	ΔG° (kJ/mol)	ΔH° (kJ/mol)	ΔS° (J/mol)	R^2^
C.I. Direct Black 80	300	−20.117	−2.619	58.330	0.997
	310	−20.704			
	320	−21.286			
	330	−21.867			

**Table 5:** Comparison of dye removal rates by our biomass with data from the literature.

No	Biosorbent	Plant Part	Removal%	q_e_ (mg/g)	Reference
1	*Bagassa guianensis* Aubl	Stem	85	0.71	[57]
2	*Xanthium italicum*	Leaf	95	1.59	[58]
3	*Citrus sinensis*	Peel	14.92	1.49	[59]
4	*Nephelium lappaceum* L.	Peel	80	108.69	[60]
5	*Punica granatum*	Peel	81.35	68.40	[61]
6	*Cocos nucifera* L.	Mesocarp	85	7.28	[57]
7	*Zygophyllum gaetulum*	Stem	97.08	97.61	Current study

**Table 6:** Characteristics of C.I. Direct Black 80.

Dye	Class	Family	Chemical Formula	Molar Mass (g/mol)	λ_max_ (nm)
C.I. Direct Black 80	Direct dye	Azoic	97.08	908.8	599.8

## Data Availability

All relevant data are within the paper.
